# Challenges in Identifying Sites Climatically Matched to the Native Ranges of Animal Invaders

**DOI:** 10.1371/journal.pone.0014670

**Published:** 2011-02-09

**Authors:** Gordon H. Rodda, Catherine S. Jarnevich, Robert N. Reed

**Affiliations:** Invasive Species Science, U.S. Geological Survey Fort Collins Science Center, Fort Collins, Colorado, United States of America; University of Zurich, Switzerland

## Abstract

**Background:**

Species distribution models are often used to characterize a species' native range climate, so as to identify sites elsewhere in the world that may be climatically similar and therefore at risk of invasion by the species. This endeavor provoked intense public controversy over recent attempts to model areas at risk of invasion by the Indian Python (*Python molurus*). We evaluated a number of MaxEnt models on this species to assess MaxEnt's utility for vertebrate climate matching.

**Methodology/Principal Findings:**

Overall, we found MaxEnt models to be very sensitive to modeling choices and selection of input localities and background regions. As used, MaxEnt invoked minimal protections against data dredging, multi-collinearity of explanatory axes, and overfitting. As used, MaxEnt endeavored to identify a single ideal climate, whereas different climatic considerations may determine range boundaries in different parts of the native range. MaxEnt was extremely sensitive to both the choice of background locations for the python, and to selection of presence points: inclusion of just four erroneous localities was responsible for Pyron et al.'s conclusion that no additional portions of the U.S. mainland were at risk of python invasion. When used with default settings, MaxEnt overfit the realized climate space, identifying models with about 60 parameters, about five times the number of parameters justifiable when optimized on the basis of Akaike's Information Criterion.

**Conclusions/Significance:**

When used with default settings, MaxEnt may not be an appropriate vehicle for identifying all sites at risk of colonization. Model instability and dearth of protections against overfitting, multi-collinearity, and data dredging may combine with a failure to distinguish fundamental from realized climate envelopes to produce models of limited utility. *A priori* identification of biologically realistic model structure, combined with computational protections against these statistical problems, may produce more robust models of invasion risk.

## Introduction

In this introduction we first establish that climate matching is a scientific activity with large public policy implications, using the example of the python. Second, we give evidence that scientific uncertainty over the optimal method for characterizing climate is a major contributor to the controversy. Third, we outline a crucial conceptual issue that distinguishes different modeling approaches to identifying potential areas of invasion. This conceptual issue is sometimes characterized as fundamental versus realized climate space and sometimes characterized as “transferability.” We then outline two other key areas of modeling controversy (overfitting, and model validation), as resolution of these key issues is highly sensitive to model conceptualization. Finally, we outline the scope of our analysis.

**Figure 1:**
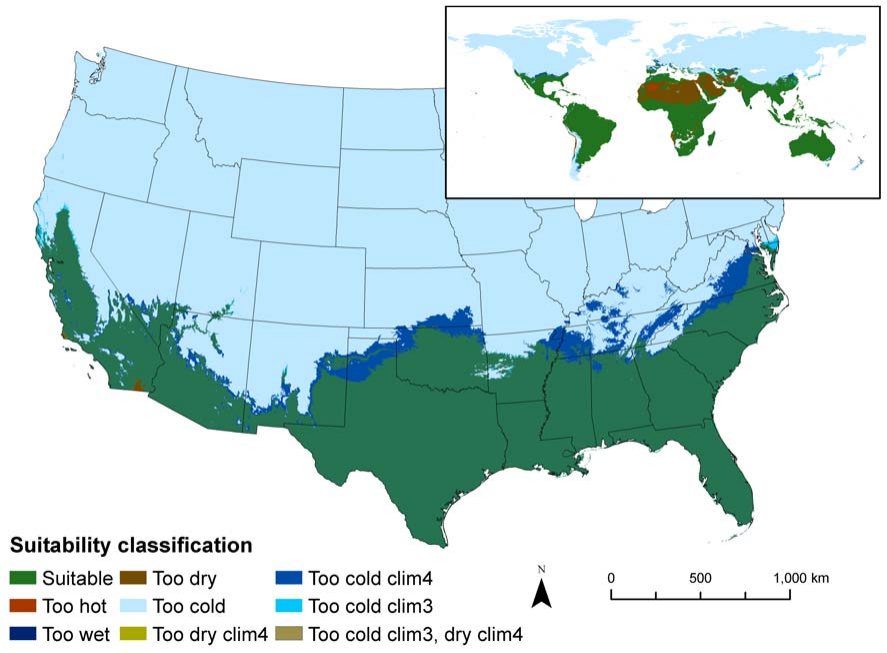
Areas matching the climate envelope expressed by *P. molurus* as detailed by Rodda et al. [**2**]. The computation was based on the snake's native range under two hypotheses of hibernation duration: clim3 (assumed duration of hibernation 3 months) and clim4 (assumed duration of hibernation 4 months). The original map was of the United States only, created using Daymet climate data (http://www.daymet.org; [69]) while the global inset was created using the WorldClim data at 30 arc-second resolution.

In 2008 the U.S. Fish and Wildlife Service (USFWS) solicited advice from the general public on the potential merits of restricting importation to minimize risk of invasion of the U.S. by nine exotic species of giant constrictor snakes [1], including the Indian Python (*Python molurus*), best known through sales of the Burmese subspecies, *Python molurus bivittatus*. At about the same time, we published results of our analysis of the areas of the U.S. that are climatically matched to the native range of the Indian Python [2], henceforth simply “Rodda et al.” For reference, the key map from that work is reproduced here as Fig. 1. The publication of our map and the USFWS Notice of Inquiry were connected in the sense that USFWS had joined the U.S. National Park Service in funding our U.S. Geological Survey (USGS) study. Understandably, some affected members of the public perceived our work as interagency collaboration in support of regulation of trade in giant constrictors, though USGS had no policy position on invasive species regulation, and we were under no pressure, either imposed by the funding sources or self-imposed, to support regulation, or bias the size or extent of the U.S. area that climatically matched the python's native range. We provided the climate match to inform the discussion.

**Figure 2:**
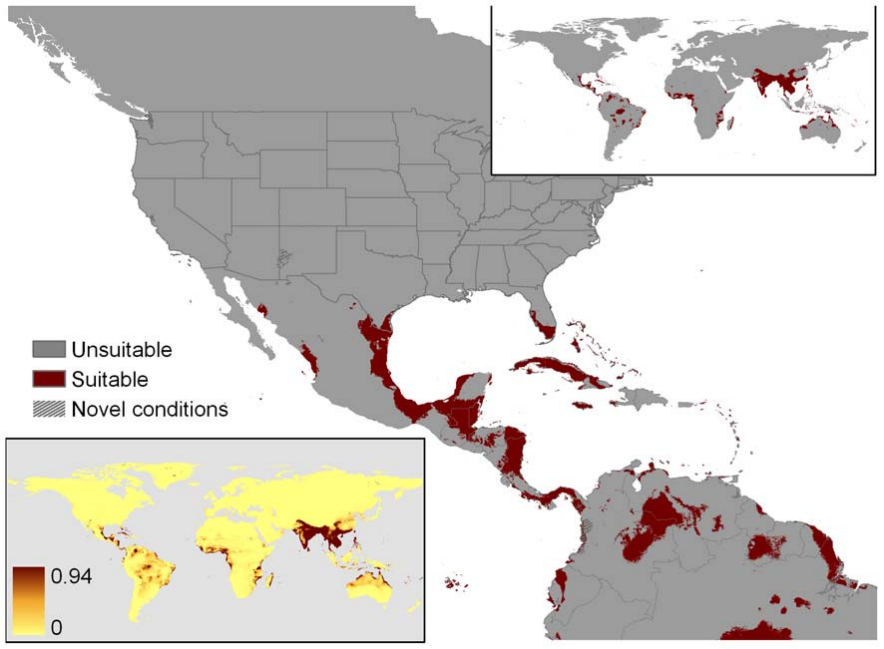
Our recomputation of MaxEnt match for Pyron et al.'s original 90 locations, using worldwide background (Overfit-Global-90 points). Novel condition localities are stippled gray. The upper inset is a global projection using the threshold adopted by Pyron et al. (minimum training presence). The lower inset portrays suitability scores by gradations of red color, where intensity increments are set by the standard deviation of training point suitability scores.

Pyron et al. [3], henceforth simply “Pyron et al.”, countered with an alternate map showing areas of the U.S. that climatically matched the python's native range; their map was embraced by opponents of regulation (e.g., [4]) because it showed a much smaller area of climatic agreement (Fig. 2). Indeed, Pyron et al. concluded that “The Burmese python is strongly limited to the small area of suitable environmental conditions in the United States it currently inhabits…” They also averred, “The proposed expansion of the python into the continental United States would require an expansion of the actual tropical marshland habitat comprising most of the Everglades, not simply the presence of similar temperature and precipitation conditions.” If either of these claims were true, no further areas of the U.S. would be at risk of colonization, and regulation of U.S. trade in this species would be largely moot. Although Pyron et al. did not expressly tie their climate match to policy, they did lay claim to the policy high ground by asserting that, “The alarmist claims made by USGS could potentially hamper scientific discourse and inquiry into the problem, especially with regard to policy-making.”

The notice of inquiry and subsequent proposed rulemaking generated a substantial public response, with a large number of comments received (55,600), and most of the criticism focused on the climate matching result for one of the nine species under consideration, the Indian Python (Art Roybal, U.S. Fish and Wildlife Service, 2010 pers. comm.). The intensity of the public reaction documents that climate matching can be a key element in establishing environmental policy, and that differences among approaches to climate modeling are critical for evaluating the scientific basis for the policy. One element of this controversy is the herpetological facts that were the basis for the models. In these, Rodda et al. and Pyron et al. did not noticeably differ and the herpetological facts will not be discussed further. Another element of the controversy is the modeling approach, for which the two teams took divergent approaches: Rodda et al. adopted a climate suitability algorithm based on first principles, and Pyron et al. used a statistical tool to discover a climate suitability algorithm. Ideally, one would have some method for validating the projections, but there is no obvious way to validate the likelihood of a hypothetical event. Furthermore, the validity of these specific models might rest on factors unique to the Indian Python, and therefore be of limited interest. More generally, we can evaluate the internal validity of the climate matching process. The *general* framework of the climate matching process was the same for both teams.

Both Rodda et al. and Pyron et al. relied on the assumption that the geographic boundaries of the species' native range offer insight into the boundaries of the species' climate envelope. For both studies, step 1 was linking native range geographic space to climate space. Having estimated the boundaries of the species' climate envelope, step 2 was projecting from inferred climate space to inferred geographic space, in this case to geographic areas where the species might invade. Both studies followed this two-step paradigm based on the native range distribution. Where the two approaches differed was in how best to choose the axes upon which to delineate the climate envelope boundaries. Rodda et al. chose axes based on their interpretation of the key ecological factors; Pyron et al. used an automated statistical algorithm (Maximum entropy modeling or program MaxEnt [5], [6]) to identify the multivariate correlations between 19 climate axes and climate conditions present at 90 geographic localities within the species' native range. Pyron et al. asserted that their model represented “ecological niche modeling” whereas ours did not. In actuality we both used the classical two step paradigm for inferring climate constraints, but differed in the algorithm with which the key climate axes were identified. We do not believe that either approach characterizes “niche.”

MaxEnt has been used for a very large number of species [7]–[11], and is the most accessible tool for non-specialists. Thus, rather than focus on the specifics of the Pyron et al. study, we here undertake a critique of the conventional (default settings) invasive animal species application of MaxEnt, with the objective of refining climate matching in general. However, our observations are intended neither to critique other applications of MaxEnt (e.g., habitat suitability mapping) nor to apply to other climate matching situations (e.g., animal range expansion, plant species). We recognize that climate matching for invasive species is a young science and current approaches, including ours, will be improved over time. We make no claim that any approach is flawless, but hope to propel improvement by pointing out the flaws that need resolution. Peterson [12] lists nine uses for species distribution or ecological niche models, of which our remarks apply directly to only one of these, the prediction of species' invasions. Our concerns apply most forcefully to inferences involving transfer of climate associations from one region or continent (usually the species' native range) to another (typically a prospective invasion range). Related issues arise when transferring inference from one temporal context to another (e.g., climate change). Our remarks specifically do not apply to plants (which lack behavioral options for local climate adaptation), use of MaxEnt for geographic interpolation (no transferability required), application to range shifting species [e.g.], [13,14], or execution of MaxEnt with different (i.e., customized) settings. We also recognize that mechanistic climate matching models [e.g.], [13]–[16] offer dramatic advantages over correlational models (e.g., Rodda et al., Pyron et al.), but mechanistic models may not be available for the screening of thousands of potential invasive species, because the requisite species-specific knowledge does not exist. Below we present concerns about the rote application of MaxEnt with regard to: 1) conceptualization of climate matching for the purpose of invasive species risk assessment, 2) the statistical approach taken when building and testing MaxEnt climate matching models, and 3) assumptions made by Pyron et al. and many other rote MaxEnt users with reference to their choices when selecting presence and background localities.

Having established that climate matching is a key tool for environmental policy making, and that MaxEnt is a key tool for climate matching, we address a conceptual issue, variously referred to as *fundamental* versus *realized* climate space, or “transferability.” This issue is crucial because MaxEnt, as it is conventionally applied, quantifies realized climate space, whereas the geographic area at risk of invasion is associated with the fundamental climate space. To fully understand this issue we need to explore the distinction between fundamental and realized *niche* space [17], [18]. As is frequent practice, however, we are referring solely to climate factors, which are only one component of niche. In our view, the fundamental climate space delineates the climatic conditions that could be occupied by a species if climate were the only limiting factor, and the realized climate space is the range of climate conditions that are actually occupied. Historical, access, non-climate abiotic, and a panoply of biotic factors can preclude occupancy of portions of fundamental climate space [19], a point ignored by many MaxEnt modelers such as Pyron et al., but already clear to Darwin ([20], p. 137): “But the degree of adaptation of species to the climates under which they live is often overrated. We may infer this from our frequent inability to predict whether or not an imported plant will endure our climate, and from the number of plants and animals brought from warmer countries which here enjoy good health. We have reason to believe that species in a state of nature are limited in their ranges by the competition of other organic beings quite as much as, or more than, by adaptation to particular climates.”

**Figure 3:**
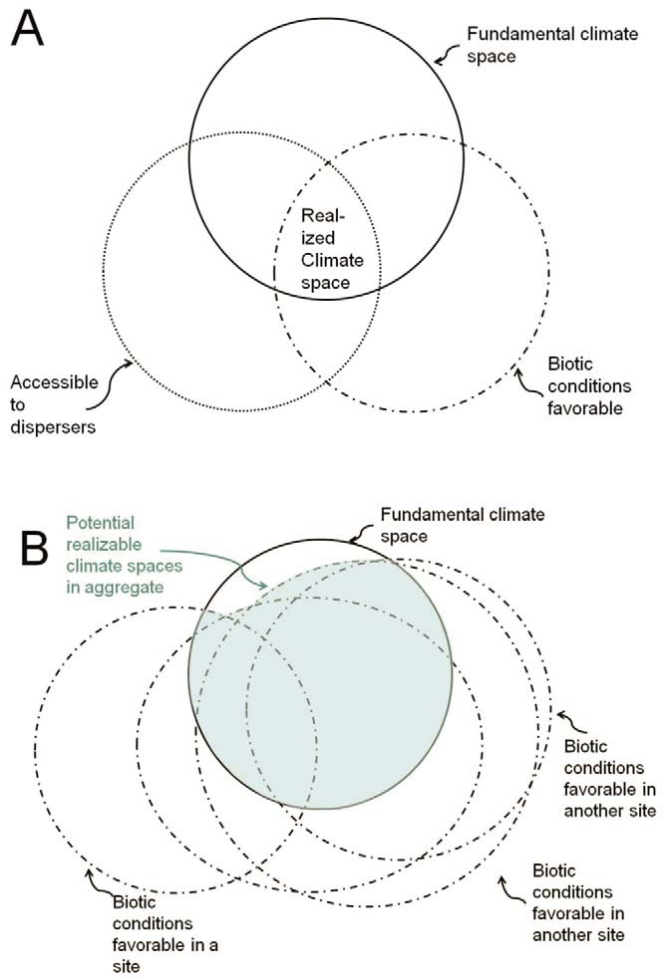
Our concept of the relationship between fundamental climate space and realized climate space. The fundamental climate space is shown by a solid line and represents conditions for which only climate is limiting. A. With reference to native range. See text for further discussion. B. With reference to prospective introduction localities (blue zone). The limitations associated with access disappear when one is considering all possible localities where a species might be introduced by human agency.

Darwin established that there is a difference between where a species does occur (realized climate space) and where an invasive species might occur if freed from other constraints (fundamental climate space). This distinction is often represented by a Venn diagram, with several different versions in print [e.g.], [ 11], [17,21]. However, none of the published versions represent our views precisely, so we created our own, for clarity (Fig. 3). Our Venn diagram of the native range (Fig. 3A) considers the overlap between three sets of conditions: climatic conditions ( = fundamental climate space), biotic conditions, and accessible areas (e.g., not separated from occupied native range by a dispersal barrier such as salt water). Hutchinson's original formulation of niche [22] distinguished only biotic from abiotic conditions. By restricting our concern to only *climatic* abiotic conditions, we run the risk of omitting consideration of non-climatic abiotic conditions; for example, the availability of abiotic refugia such as rock crevices or subterranean hibernacula. Thus a complete rendition of the factors constraining occupied climate space would need to include an additional set representing limiting non-climatic abiotic conditions. For simplicity, we have omitted this set from our diagram, but are mindful of the importance such factors could play in the viability of populations, including that of the focal species.

The degree of overlap between the conditions represented in our Venn diagram is contingent on the geographic location under consideration; for Figure 3A this is the native range. In our view, the boundaries of the fundamental climate space are usually stable over a management time frame (decades); in this sense they are fundamental. Fundamental niche attributes tend to be evolutionarily conserved [12], [23], and therefore evolution of the fundamental climate space boundaries will ordinarily occur slowly, over time frames longer than is relevant for invasive species management policy. The other two circles are highly contingent, moving their shape and position in reference to differing focal locations [24]. The biotic conditions associated with a single potential introduction site (e.g., a given dashed circle in Fig. 3B) differ in overlap with the fundamental climate space from that present in the native range (Fig. 3A), because different biotic conditions prevail in different geographic areas.

We treat the native range as occupying only the union of all three conditions in Fig. 3A (Fundamental Climate Space∩Biotically Favorable∩Accessible), which we call the realized climate space [22]. The realized climate space in a Venn diagram of a specific introduced range would also be the triple union (not illustrated), but the region accessible to dispersers in a single introduction site might be rapidly growing over time as the population spreads (i.e., the boundaries of the access circle may be very dynamic [25]).

Our conceptualization differs from some others in that we take no position on the importance of competition over other biotic factors [21]. Under typical conditions of human-aided transport (e.g., in the absence of hybridogenesis), we would expect translocation to not affect the fundamental climate space [11], but to alter the realized climate space, which would be prone to expansion as a result of expanded access (by definition, human translocation is manifest in a relaxation of access barriers), and altered, often more permissive, biotic conditions [26], [27].

One policy challenge to a regulator of prospective invasive species is to determine what geographic space is potentially occupiable (Fig. 3B). We concur with Peterson [12], Jimenez-Valverde et al. [24], and B. Phillips et al. [13] that such areas are best estimated by matching of the fundamental climate space to the prospective location. This is the viewpoint expressed by Darwin, though he did not use the newer terminology (“fundamental” and “realized”).

Instead of discussing this issue in terms of fundamental and realized climate spaces, many observers refer to “transferability” of the climate match from a species' native range to an introduced range [27]–[30]. That is, researchers model the realized climate space in one or more parts of the world and ask whether the inferred climate envelope “transfers” to the realization of the fundamental climate space that has occurred elsewhere. The few such studies have produced inconsistent records of transferability [27], [31], and have been generally unfavorable in the few studies of reptiles or amphibians [9], [11], [16]. Although there have been examples of birds whose introduced population's equilibrium range limits reflect a climate envelope that was smaller than one similarly derived from its native range [32], the majority of examples, especially of herpetofauna, reflect the converse: introduced ranges reflect a greater climatic range than was found in the native range [16], [33], [34]. The general pattern of greater climatic scope in the introduced ranges has led some observers to seek a general explanation based on more favorable biotic conditions (fewer predators, less disease, fewer parasites, etc.) in the introduced range [26]. Constraining possibilities include the absence or presence of dispersal barriers in the introduced range; failure to model a limiting factor that applies in both ranges, but is more geographically limiting in one of the ranges; and an introduced range that is not at ecological equilibrium (spread still progressing).

This discussion of realized and fundamental climate spaces highlights the problems of verification of climate matching models. If one were to withhold a portion of the native range points to validate one's model of the realized climate space, and if one were to target in the model fit a balancing of geographic errors of commission (unoccupied range judged suitable) and omission (native range judged unsuitable), as Pyron et al. and many others have done (see [35]), one might obtain a relatively “accurate” model, but it would be of the wrong (i.e., realized) climate space. An impressive test AUC (Area Under the receiver operating characteristic Curve) is no indicator of model value if you're modeling the wrong target. Transferability is more likely to be robust if the *fundamental* climate space is modeled well (axes represent true ecological drivers), or in the unlikely event that biotic and dispersal factors present in the native range are functionally and geographically equivalent to those in the introduced range and the covariance structure among the climate axes is unchanged between native and introduced ranges [25], [28].

Overfitting of the realized climate space is a problem often cited with highly parameterized species distribution models [25], [28], [36], [37] such as MaxEnt models created with default parameter settings. Overfitting will induce underprediction when the climate model is applied to new geographic locations. Overfitting also affects the application of a climate model to novel conditions (those climates not considered in the calibration of the original model). With overfitting, additional geographic areas are subject to the “novel” descriptor as additional climate dimensions (axes) are used in the model. However, statistical overfitting is usually referenced only to the *realized* climate space; overfitting metrics do not ordinarily consider overfitting with reference to the larger, fundamental climate space.

MaxEnt in the default settings uses “regularization” parameters that are optimized on the basis of external calibration data, not the actual data set used, to constrain overfitting, but the target for these “rule-of-thumb” regularization parameters is the training (i.e., realized) climate space not the fundamental climate space [38]. In the version of MaxEnt we used (3.2.3a), the rules-of-thumb for regularization parameters were based on twelve species (one frog, one reptile, three birds, seven plants: [38]) with 11 to 13 environmental variables and numerous well-behaved locality data (more regularization may be needed for more complex models, such as the 19 environmental variables used by Pyron et al., or weaker locality data). MaxEnt's regularization parameter settings based on a mix of plants and animals might or might not be appropriate for pythons, or for a particular set of localities such as clustered point locations. Thus it would be useful to have a mechanism for applying regularization to the actual data used.

One possible data-specific test is based on splitting the data between training and test fittings [39]; overfitting of the *realized* climate space should result in a lower accuracy for the test data relative to the accuracy associated with the training data.

Correct fitting of the realized climate space will result in an equivalent accuracy for the test data, but it has to be recognized that even an *optimal* fitting of the realized climate space will result in an underprediction of the fundamental climate space that is of interest. For this reason, Jimenez-Valverde et al. [24] concluded that simple models (fewer parameters, simpler relationships) should be favored over complex ones (more parameters, more complex functions: overfit) for modeling potential invasive distributions. Therefore the penalties for underfitting and overfitting are asymmetric in the case of projecting potential invasive ranges. Underfitting of the native range (realized climate space) will more closely approximate the fundamental climate space than will the optimal fit, whereas overfitting of the native range will underpredict the realized climate space and err even further from accurately predicting the fundamental climate space that is of interest for projecting potential invasive ranges. As we show below, Pyron et al. grossly overfit the realized climate space, thereby underpredicting both realized and fundamental climate space. This phenomenon is likely to occur with many default applications of MaxEnt to invasive species climate matching.

We also discovered that Pyron et al. used several localities for the wrong species, and chose background points from a global pool, rather than true absences or the regional background recommended by the developer of MaxEnt [30], [34], [40]. Such data errors appear often in data sets; by comparing MaxEnt output with and without correction we explore MaxEnt sensitivity to their occurrence. To subject our rule-based method for climate matching to a similar challenge of input variation, we estimated the climate space captured by a range of sample sizes with our algorithm. To address Pyron et al.'s criticism that we erred by including empirical climate data from native range weather stations rather than using modeled climate for point presences, we also consider two presence point data sets using modeled rather than empirical climate statistics.

## Materials and Methods

**Figure 4:**
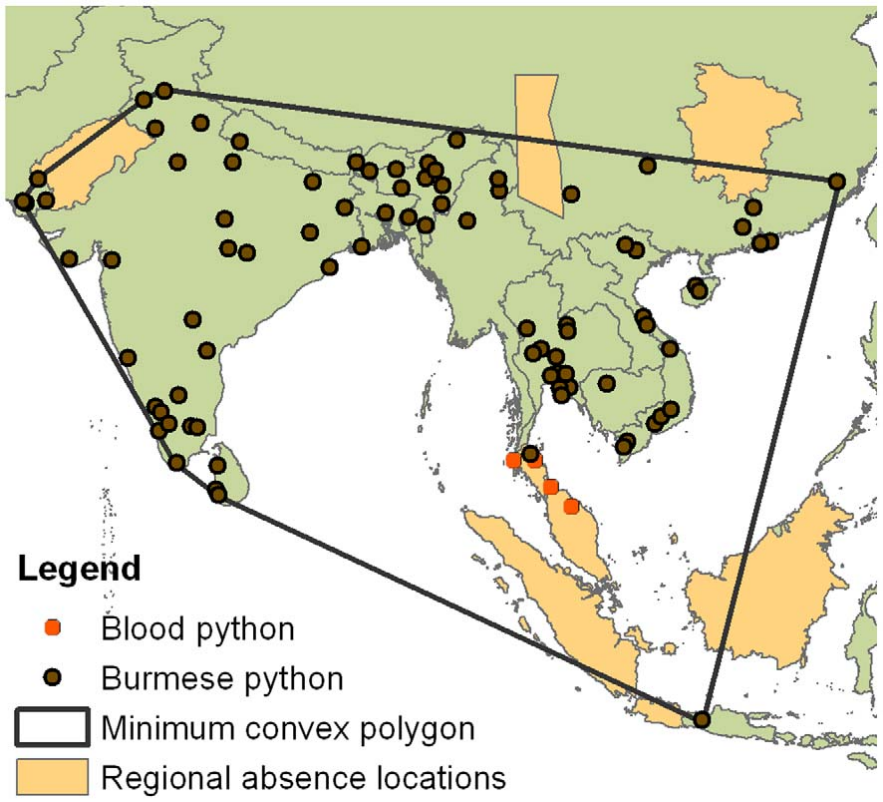
Backgrounds and locality points used for Maxent models.

In this work we recomputed MaxEnt models using the published protocol of Pyron et al., with the exceptions stated below. However, we were unable to exactly duplicate their results in all details, despite contact with the authors to determine what settings may have differed from those used in their paper. However, only a very sharp-eyed observer will be able to detect any discrepancies, and they do not affect the issues raised. We followed Pyron et al.'s lead in using the least-probable training point likelihood (“minimum training presence”) to set the threshold for discriminating suitable from non-suitable habitat. Except for two specified model runs, our input localities differed from that of Pyron et al. only in that we used 86 rather than 90 localities. The four excluded localities were taken by Pyron et al. from Nabhitabhata and Chan-ard [41], but in that document they are labeled as localities for a different python, the Blood Python (*Python brongersmai*). Thus our use of 86 rather than 90 localities simply corrected an input error (included points and omitted points shown in Fig. 4). The other key change we made regarded the choice of background conditions, often discussed as “pseudo-absences” (MaxEnt developer Phillips (pers. comm.) rejects the characterization of “absences,” as MaxEnt assumes that background conditions include true presences;). We considered three alternative suites of background conditions, utilizing the exact same background points in each set of models for the three suites.

The first set of two models (worldwide background) followed Pyron et al. The second set, of three models, used the conventional choice of background localities from the region of the presences (here defined as the minimum convex polygon (MCP) of Pyron et al. localities plus 2 pixels (2.5 min resolution; Fig. 4)). The conventional MCP choice of background localities has been criticized for minimizing the contrast between presence and absence, especially for wide-ranging species of low detectability, such as our subject. For this reason, Lobo [42], [43] recommended selecting background from areas that are immediately adjacent to occupied habitat but are known to be unoccupied. To determine the impact of such a choice on MaxEnt's background selection, we adopted this rationale for our third choice of background (one model), which were taken from the Thar Desert, eastern China north of the known range, central China west of the known range, the Malay Peninsula south of the Isthmus of Kra, Borneo, Sumatra, and small islands offshore of those large Indonesia islands (Fig. 4).

Using the conventional MCP background choice, we computed three MaxEnt models, based on: 1) 86 localities modeled using the default regularization setting, 2) 90 localities using increased regularization, as determined by the application of the small sample corrected variant (AICc) of Akaike's Information Criterion (AIC) recommended by Warren and Seifert [39], and 3) 86 localities modeled using the same AIC-based regularization. Contrasting the latter two models allowed us to assess the degree to which reduced-complexity models remained sensitive to input variation, and how variation in reduced-complexity models altered identification of key climate axes and thresholds associated with minimum training presence. We projected these three MCP models to the globe, and had MaxEnt calculate locations with novel conditions (i.e., locations with climate outside the range of that covered by the presence and background locations used to develop the model) via the MESS analysis tool [14].

To estimate the precision of each of the six MaxEnt models, we ran each 25 times, withholding a different 10% of the localities each time. For direct comparison to Pyron et al., we also ran a single run of each model, specifying the training and test data locations to ensure consistency. We judged a climate axis to be “important” if its percent contribution exceeded 10%, and we evaluated the suitability of each climatic condition on the basis of the marginal response curves. To assess whether the alternate metric of climate variable importance - permutation importance - was consistent with the pattern exhibited by percent contribution, we computed r^2^ for the correlation between “important” variable weights in these two metrics (we omitted variables which were rated unimportant with both metrics), separately for overfit and AICc constrained models. For each model we counted parameters using the algorithm of Warren and Seifert [39].

We computed the correlation matrix of the 19 climate axes used by Pyron et al., based on the climatic conditions prevailing at 5000 random localities within the native range region.

In addition to recomputing MaxEnt with alternate presence localities and alternate selections of background, we recomputed our rule-based model using alternate native range climate inputs, and we tabulated our climate space under a variety of reduced sample sizes to judge the sensitivity of our method to small samples. Our alternate native range climate inputs were selected to match the localities and procedure of Pyron et al., who used modeled climate from museum collection localities, a method they judged superior to our use of empirical climate records from areas within the native range (but not demonstrably occupied by pythons). However, as Pyron et al. had substantially fewer localities than was used in our original model, we considered both the small Pyron et al. locality list and an augmented list to assess the sensitivity of this result to a range of sample sizes. For the small list of localities (84 points) we omitted two additional questionable localities from Pyron et al.'s 86, one of which simply failed to generate usable climate data from the WorldClim dataset (available at http://www.worldclim.org; [44]); the other locality was outside of the known range of the Indian Python (south of Isthmus of Kra), and may represent a recent range extension, a human translocation, a recording error, or inaccurate characterization of the native range. For the larger list (98 localities) we added 14 localities at the northern and western fringes of the native range, which were poorly represented in the Pyron et al. data set. The additional localities were from the literature [45]–[49] or from specimens at the California Academy of Sciences.

To judge the sensitivity of our rule-based method to reductions in sample size, we computed the relative amount of climate space that would have been detected by our method had our sample been a random subset of the original 149 localities [2]. Subsequent to the original analysis, we identified 2 additional suitable localities; thus our estimate of the sensitivity of our method to sample size was based on ten random draws for each decile of the 151 localities, with area computed in units of 0.1 log10(Precipitation in mm/mo) and degrees C.

## Results

**Figure 5:**
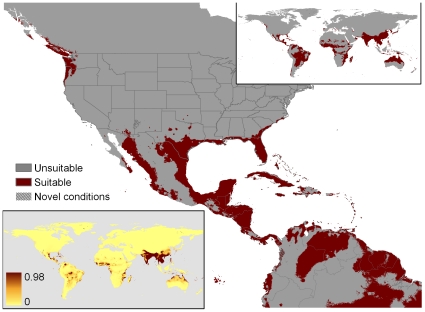
Our recomputation of MaxEnt match based on Pyron et al.'s 86 locations using worldwide background (Overfit-Global-86 points). The localities distinguishing the input data set for this figure from that of Fig. 2 were the four sites occupied by a different species (Blood Pythons, as indicated in Fig. 4). Other mapping conventions as in Fig. 2.

**Figure 6:**
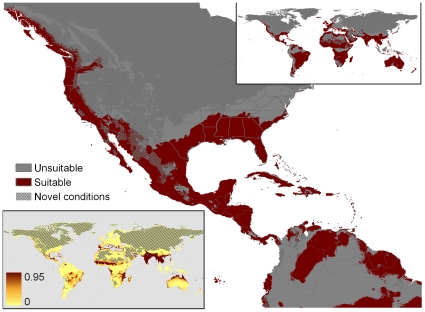
Our recomputation of MaxEnt match based on Pyron et al.'s 86 locations using regional absences (Overfit-Regional absences-86 points). Other mapping conventions as in Fig. 2.

**Figure 7:**
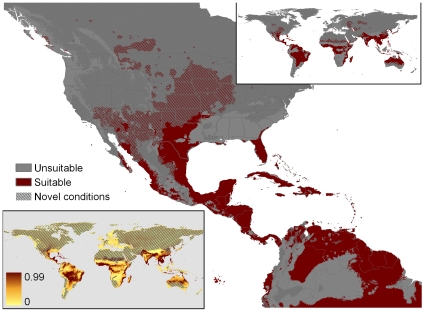
Our recomputation of MaxEnt match based on Pyron et al.'s 86 locations using for background the minimum convex polygon around the native range (Overfit-MCP-86 points). Other mapping conventions as in Fig. 2.

**Figure 8:**
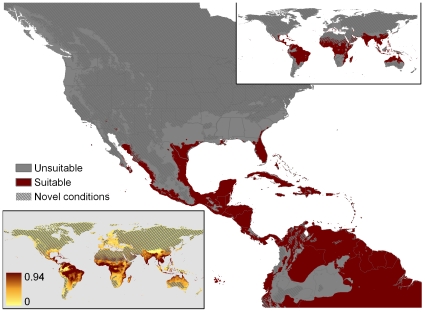
Our AICc-constrained recomputation of MaxEnt match based on Pyron et al.'s 90 locations using for background the minimum convex polygon around the native range (AICc-MCP-90 points). Other mapping conventions as in Fig. 2.

**Figure 9:**
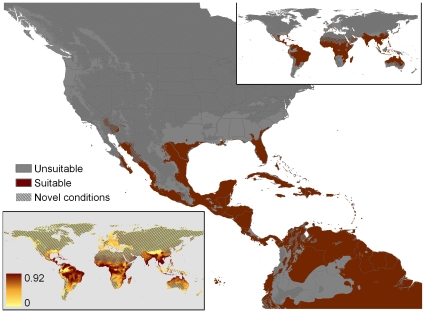
Our AICc-constrained recomputation of MaxEnt match based on Pyron et al.'s 86 locations using for background the minimum convex polygon around the native range (AICc-MCP-86 points). Other mapping conventions as in Fig. 2.

**Figure 10:**
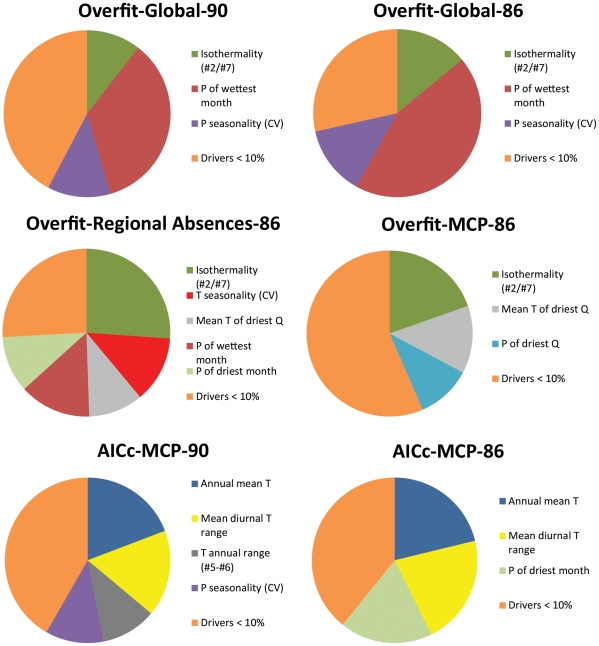
Important climate variables for each of the six MaxEnt models. Variables contributing less than 10% to the model are aggregated in “Drivers<10%.”

The six MaxEnt model results are summarized in Table 1; the geographic projections for the six models are in Figs. 2, 5, 6, 7, 8, 9, and a graphical summary of important climate variable contributions is in Fig. 10. The original Pyron et al. model (Overfit-Global-90 points), replicated 25 times, produced a minimal but plausible geographic match to the U.S. (Fig. 2), minimal match to tropical areas of the world outside of the native range (Fig. 2 insets), geographic evidence of overfitting (deeply fragmented matches in much of the tropical world), high parameter counts (approaching the number of localities used as input: Table 1), an exemplary test AUC (0.971), and a very low minimum training presence (0.092). The important input variables (fig. 10) were isothermality (11%; lower daily range more suitable), precipitation of the wettest month (35%: wetter sites more suitable), and precipitation seasonality (12%: more variable rainfall was more suitable).

**Table 1:** Overview of MaxEnt models considered.

Name	Over-fit?	Back-ground	Local-ities	Beta	Runs	Para-meters	Minimum training presence	AUC train	AUC test
Overfit-Global-90 pts	Yes	Global	90	1	25	62 (5)	0.092 (0.025)	0.984 (0.001)	0.971 (0.010)
				**1**	**1**	**66**	**0.101**	**0.985**	**0.965**
Overfit-Global-86 pts	Yes	Global	86	1	25	59 (4)	0.013 (0.019)	0.982 (0.001)	0.973 (0.011)
				1	1	60	0.008	0.982	0.971
Overfit-Regional absences- 86 pts	Yes	Regional Absences	86	1	25	50 (5)	0.003 (0.001)	0.988 (0.002)	0.976 (0.029)
				1	1	57	0.002	0.990	0.972
Overfit-MCP-86 pts	Yes	Native Range MCP	86	1	25	56 (5)	0.159 (0.021)	0.816 (0.007)	0.702 (0.074)
				1	1	52	0.156	0.829	0.632
AICc-MCP-90 pts	No	Native Range MCP	90	3 (0.7)	25	13 (2)	0.237 (0.230)	0.747 (0.010)	0.711 (0.077)
				4	1	10	0.261	0.739	0.739
AICc-MCP-86 pts	No	Native Range MCP	86	4 (1.2)	25	10 (2)	0.222 (0.239)	0.718 (0.010)	0.624 (0.093)
				3	1	14	0.184	0.748	0.748

The **bolded** values represent the model presented in the original Pyron et al. work; the other single run models are provided for direct comparison. “MCP” indicates a minimum convex polygon surrounding the native range. “Overfit?” indicates use of default regularization in lieu of the small sample corrected AICc (Akaike's Information Criterion) method of Warren and Seifert (2010). “Localities” references number of native range geographic localities used, and indicates inclusion/exclusion of the four Blood Python points. “Beta” indicates the regularization multiplier used. Parenthetical values are standard errors.

Removal of the four Blood Python localities (Overfit-Global-86 points) produced a radically different climate match to the U.S. (Fig. 5). Whereas inclusion of the Blood Python points had produced a MaxEnt model that matched only the extreme southern tips of Florida and Texas (Fig. 2), exclusion of the 4 erroneous points led to matches throughout the Gulf Coast, and the Atlantic coast north to the Outer Banks (North Carolina), as well as climate-matched localities on the Pacific coast from northern California northward to Alaska. The model found climatically suitable inland sites in Arizona, New Mexico, Texas, and Oklahoma. The match also included some surprisingly temperate zones in the southern Andes (Fig. 5 insets). Like the original model, Overfit-Global-86 points had minimal match to tropical areas of the world outside of the native range (Fig. 5 insets), geographic evidence of overfitting (fragmented matches in much of the tropical world), high parameter counts (Table 1), an exemplary test AUC (0.973), and an extremely low minimum training presence (0.013). The important input variables (fig. 10) were isothermality (14%; intermediate daily ranges more suitable), precipitation of the wettest month (44%: wetter sites more suitable), and precipitation seasonality (12.2%: more variable rainfall was more suitable). Because all of the world's terrestrial climates were present in the background training conditions, MaxEnt identified few novel conditions for these models (Figs. 2, 5).

The Overfit-Regional absences-86 points model had a substantially larger geographic projection to the U.S., with appreciable portions of all coastal states (and inland as far as Arkansas) from North Carolina to Alaska (Fig. 6). The model did not produce any climate matches in the interior, and novel conditions existed throughout the interior (Fig. 6). The geographic match included some remarkably temperate coastal localities (extreme Southern Andes, Norway, Iceland, Aleutians), but the tropical areas were less fragmented than in the preceding two models, suggesting reduced overfitting. Nonetheless, it had a high parameter count (Table 1), a high test AUC (0.976), and an even lower minimal training presence (0.003). Three of the five important climatic variables did not appear in the global models (temperature seasonality: 14%, aseasonal areas more suitable; mean temperature of the driest quarter: 12%, intermediate temperatures favorable; precipitation of the driest month: 12%, least precipitation more suitable), but isothermality (29%, low daily ranges more suitable) and precipitation of the wettest month (16%, wetter sites more suitable) were again found to be important.

The three remaining models all used for background the minimum convex (MCP) polygon surrounding the native range. Overfit-MCP-86 points had an extensive area of suitability in the U.S. (Fig. 7), primarily interior sites (and peninsular Florida and coastal British Columbia). However, most of these interior sites exhibited novel conditions, reducing certainty about their suitability. Locations with novel conditions were identical for all the MCP models (i.e., extensive in interiors of subtropical and temperate continental areas). Global matches were mostly tropical and coastal, but included a large area in the interior of South America, and small unexpected patches in places like Japan and Denmark. Geographic fragmentation was intermediate, the parameter count was again high, the mean test AUC was substantially lower than the preceding models (0.702), and the minimum training presence was much higher (0.159). Isothermality was the most important climatic variable (20%, low daily ranges more suitable), mean temperature of the driest quarter reappeared (13%, low and intermediate values equally suitable), as well as a new climate variable: precipitation of the driest quarter (11%, all except the lowest values suitable).

AICc-MCP-90 points produced a relatively modest fit to the U.S., largely limited to peninsular Florida and coastal Texas, with most continental interiors masked due to novel conditions worldwide at subtropical and temperate latitudes. Suitable areas included most of the wetter tropics, for which suitable blocks were mostly unfragmented (Fig. 8), as was expected given the much lower parameter count. The mean test AUC was again low (0.711), but the minimum training presence was higher (0.237). The important climatic variables had little similarity with those identified in the previous models: 19% annual mean temperature (hotter better); 17% mean diurnal temperature range (reduced variability better); annual temperature range: 11% (reduced variability most suitable), and precipitation seasonality: 11% (high variability most suitable).

AICc-MCP-86 points hardly differed from AIC-MCP-90 points in its geographic match to the U.S. or to the world (Fig. 9 and Fig. 9 inset), placement and extent of novel climates, parameter count, fragmentation, minimum training presence, and the role of the top two climatic variables. Test AUC was slightly lower (0.624), and precipitation of the driest month emerged in importance (18%, precipitation of the driest month: least precipitation most suitable) in apparent replacement for annual temperature range and precipitation seasonality.

The relationship between the two metrics of climate variable importance (percent contribution and permutation importance) was negligible for both the four overfit models (r^2^ = 0.04) and the two AIC models (r^2^ = 0.01).

**Figure 11:**
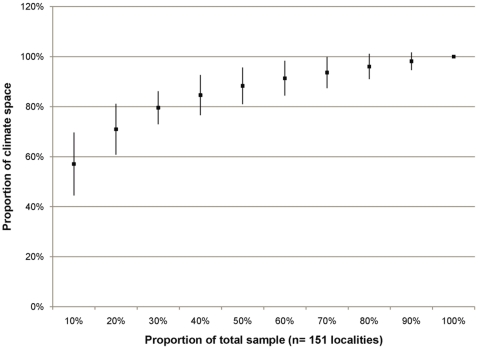
The relative climate space captured by samples of various sizes (our full sample = 100%). Shown are means of ten random draws for each decile +/− S.E.

We estimated the effect of sample size on the Rodda et al. rule-based algorithm for climate matching (Fig. 11), in which about 55% of the climate space was detected in an average sample of 10% of our localities. A sample size of 50% (75 of 151 localities) averaged 88% of the climate space of the full data set, and larger samples captured an average of at least 90%.

The 19 climate axes used by Pyron et al., were multi-collinear (Table S1). The modal r value among the 171 pairwise comparisons was in the 0.8–0.9 decile, with 28% of the available correlations in excess of 0.8. Two-thirds (115 of 171) of the r values exceeded 0.5.

**Figure 12:**
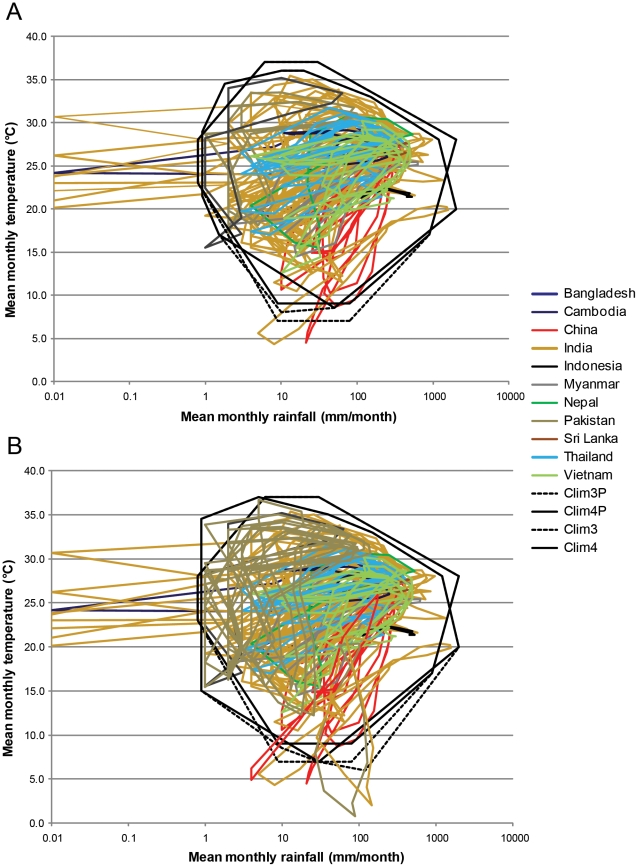
Climate space as inferred from the specimen localities and modeled climate. Climate space tabulation following the method of Rodda et al. with the assumption of three (Clim3P) or four (Clim4P) months of hibernation. The equivalent polygons derived from empirical native range climate data associated with inferred occupancy are given by the thinner lines, Clim3 and Clim4, respectively. Panel A is based on the 84 specimen localities in Pyron et al., and shows slightly reduced climate space in comparison to the 151 climate station localities computed by Rodda et al. Panel B is based on 98 specimen localities, and shows slightly greater climate space in comparison to the 151 climate station localities.

We applied two samples of documented presence localities (84 localities or 98 localities) to our rule-based algorithm for characterizing climate space in the Indian Python. As estimated with modeled climate from Hijmans et al. [44], these two sets yielded very similar climate envelopes and closely bracketed those we obtained using empirical climate data (Fig. 12) and inferred presence localities. For the models that treated the Indian Python as being capable of three months of hibernation, climate space in relation to our original computation [2] was 94% and 107% for the 84 locality and 98 locality compilations respectively. The equivalent values for four months of hibernation were 95% and 105%.

## Discussion

Our results highlight the variation among MaxEnt models with slight differences in inputs or parameter values. It is evident that no single run or single model constitutes a definitive solution [c.f. 3]. How does one go about choosing among the various iterations? In our experience, AUC is not useful for choosing among the models. For example, the first three models we considered (Overfit-Global-90, Overfit-Global-86, and Overfit-Regional absences-86) had widely divergent geographic matches to the U.S. and yet all had statistically indistinguishable test AUCs of 0.971–0.976 (Table 1). What alternate metrics could be used to distinguish the relative merits of these models? We identified four screening tools that are insufficient for careful ranking of models, but can be used to screen out unacceptable models, and could in some cases be refined into tools for relative or absolute ranking. They are as follows:

### Native range inclusion

As we indicated in the introduction, the fundamental climate space is expected to be more inclusive than the realized climate space. Thus a climate matching model that showed low suitability of any major portion of the occupied native range (see Rodda et al. Fig. 1 or Pyron et al. Fig. 1 for native range delineation) would have failed to capture the full realized climate space, much less the fundamental climate space [19]. The native range screening tool is asymmetric: false omissions of geographic space are penalized, but false commissions of geographic space are not. Pyron et al. criticized the “false” commissions associated with our model, but this reflected a misunderstanding of the relationship between realized and fundamental climate space. Three of our six MaxEnt models showed low suitability for large occupied regions of interior India (false omissions), and thus were judged to fail the native range inclusion screen (Table 2).

**Table 2:** Screening scores of MaxEnt models considered.

Name	Native Range	Minimum Training Presence	Overfitting	Eco-plausibility Test
Overfit-Global-90 pts	fail	fail	fail	pass
Overfit-Global-86 pts	fail	fail	fail	fail
Overfit-Regional absences-86 pts	pass	fail	fail	fail
Overfit-MCP-86 pts	fail	pass	fail	fail
AICc-MCP-90 pts	pass	pass	pass	pass
AICc-MCP-86 pts	pass	pass	pass	pass

Low suitability for significant parts of the species' native range earned a failing score under “Native Range.” Low suitability scores (<0.1) for occupied localities earned a failing score under “Minimum Training Presence.” Parameter counts in excess (×2) of those warranted by Akaike's Information Criterion earned a failing score under “Overfitting.” Ecologically implausible geographic matches (e.g., Scandinavia, British Columbia, Aleutians) for this heliophilic sub-tropical snake earned a failing score under “Eco-plausiblity Test.”

A consideration important to the evaluation of native range occupancy is whether existence of a specimen from a given area is sufficient proof that the area is occupied (i.e., the population is self sustaining). Sink habitats are only temporarily occupied, and yet may yield a specimen on occasion. This issue is very dependent on the taxon under consideration. Plants that are wind or bird dispersed may sprout in areas far from self-sustaining populations. Birds and marine organisms may fly, swim, or drift enormous distances from the climatically suitable ranges. However, the vagility of most reptiles and amphibians is miniscule by comparison. Having relatively limited ability to create internal thermal and hydric conditions suitable for their survival, they are extremely sensitive to climate, and refractory to crawling beyond their climatic limits. Furthermore, their limited vagility puts an upper bound on vagrant dispersal distance. Even wandering reptiles are likely to be within the pixel diameter (∼1 km) used in this study. In addition, in most terrain, climate changes on a much larger scale than 1 km. Sink habitats can be a problem associated with climate inference of some taxa (for which minimum training presence would not be an appropriate threshold), but are unlikely to be a concern with most reptiles and amphibians (sea turtles and crocodilians excepted) in their native range.

### Minimum training presence

If demonstrably occupied localities correspond to realized climate space, the discriminating power of a model can to some degree be quantified by the degree of separation between presence points (high suitability only) and the background (high and low suitability). Under ideal conditions, suitability scores for occupied habitats would have a very sharp suitability cutoff (∼square wave), which would give confidence that the correct environmental factors had been identified, and occupied localities would be uniformly characterized by high (e.g.,>0.5) suitability scores. However, some of our MaxEnt models had shockingly low minimum training presences (Table 1). The most extreme example was the Overfit-Regional absences-86 model, which associated one occupied locality with a suitability score of 0.003. This indicates that 99.7% of the suitability range was suitable for the species, evidence of an extremely poor discriminator. Similarly, the first three models scored more than 90% of the suitability range as suitable, and therefore we judged those models to fail as credible discriminators (Table 2). One could skirt this problem by arbitrarily eliminating some of the occupied localities (which was not done by Pyron et al. or Rodda et al., but is recommended by Phillips (pers. comm.), and is embraced by some researchers working on taxa with high vagility or drift potential; Pyron has also chosen (2010 pers. comm.) to reverse his earlier position on this point). We do not think that arbitrary omission of localities is appropriate for most reptiles in the invasive species context under consideration.

### Overfitting

Overfitting is discussed in more detail below, but as an initial screen for unsuitable models, we believe that Akaike's Information Criterion (AIC, in our case specifically AICc) has considerable merit and should be applied. This does not address the discrepancy between fundamental and realized climate space, but it ensures that one's top models are in a reasonable range of complexity. As a screening tool, we eliminated all models that had more than twice the number of parameters included when regularization is optimized using AIC (Table 2). In the absence of AIC computations, one can get some sense from the fragmentation observed in geographic matches to climatically uniform regions (e.g. central Amazon basin). The screenings portrayed in Table 2, however, were based exclusively on AIC-determined parameter counts, as AIC scores were available.

### Eco-plausibility test

The basic ecology of Indian Pythons is known in enough detail to understand that they are unlikely to do well in fog-bound high latitude maritime places such as Scandinavia, the coast of British Columbia, or the Aleutian Islands. It would be difficult to refine this assessment into a quantitative metric of model value, but as a screening tool it is credible to assess that three of the models did not pass the eco-plausibility test (Table 2).

The aggregate application of these four screening tools results in the retention of only two models (AICc-MCP-90, AICc-MCP-86), which were virtually identical in geographic projections, minimum training presence, and relative lack of overfitting. It is notable that these two models had relatively low AUC values, supporting Lobo et al.'s [35]; [ see also 39] assertion that AUC can be a misleading guide to model utility. There were some major differences in climate variables identified by the two plausible models, even though these two models' input differed only in the inclusion of four nearby localities.

Unfortunately, we have no assurance that appropriate application of screening tools will guarantee the removal of all erroneous models in all circumstances. The full suite of models indicated that relatively minor variations in MaxEnt presence or background localities could produce radically divergent climate matches and sharply varying identification of climate drivers. The divergence was evident both within MaxEnt models (Figs. 2, 5, 6, 7, 8, 9) and between MaxEnt models and our rule-based method (Figs. 1, 2, 5, 6, 7, 8, 9). The dramatic divergences focus attention on sensitivities in the conventional application of MaxEnt, but the analyses conducted suggest but cannot pinpoint more general reasons for the discrepancies between and among models. In the following sections we present our assessment of the likely reasons for the discrepancies, and what those reasons might mean for invasive species climate matching, organized around: 1) conceptual issues, 2) statistical concerns, and 3) the selection of presences and absences. We recognize that proper execution of a model is the responsibility of the modeler, and that software is not good or bad but useful or less so. The choices made by Pyron et al. when using MaxEnt mirror those by many other users; we leave it to readers to assess the degree to which the identified problems can be satisfactorily resolved within the options provided by MaxEnt.

### Conceptual issues

We have two major concerns about how MaxEnt and other climate space models are routinely used for the purpose of projecting potential invasion localities: 1) modeling is targeted at one (occasionally more) realized climate space(s), whereas the greatest interest lies with the more inclusive fundamental climate space; and 2) modeling is typically premised on the assumption that a single ideal climate exists for each species, and that this archetype can be discovered by tallying the central climate tendency associated with localities tied to museum specimens.

The first issue (realized versus fundamental climate space) has been discussed in the Introduction. Pyron et al. claimed (p. 2) that they were characterizing the fundamental climate space, but their methods make it clear that they were trying to characterize the climate of the native range only (realized climate space). Given that biotic factors rather than climate are believed to be the main drivers of distributional limits at low latitude [5], [50], [51], the discrepancy between the two is likely to be especially important for low latitude species.

Although the desirability of characterizing fundamental rather than realized climate space was outlined in the Introduction, a method for doing so is not clear [52]. There is an interaction between this challenge and the risk of overfitting. Overfitting reduces the projection to potential invasive localities (underpredicts), as does calibration of a model to the realized climate space rather than the fundamental climate space. Overfitting also adds axes upon which an occupied locality might be judged less suitable, lowering the minimum training presence and in that way altering the geographic projection. In this respect it is notable that the AIC-constrained models exhibited minimum training presences that were high and nearly indistinguishable (6% change: 0.222 versus 0.237), whereas the same change in input for the Overfit-Global models produced a seven-fold difference (0.092 versus 0.013) in low minimum training presences.

A strong suggestion for improved estimation of fundamental climate spaces is to include introduced ranges in the characterization of the realized climate spaces [10], [31], as illustrated in Fig. 3B. We see no disadvantage to this approach, especially as it at least doubles the sample size of realized climate spaces (from one to at least two). For species such as *Hemidactylus frenatus* that have colonized dozens of times [11], [53], the sample size of realized climate spaces can be greatly increased. Fortunately for biodiversity preservation, and unfortunately for climate modelers, many potential invasive species do not have a track record of extralimital colonization.

A limitation on the inclusion of realized climate spaces expressed by species introductions is that many introduced populations are still spreading (e.g., the Florida population of the Indian Python; see also [25], [54], [55]), or they are bounded by access limitations (e.g., the species is on an island: [56]) that limit the climate space that can be occupied. Failure of an introduced population to widen the boundaries of realized climate space is not evidence that the limits of fundamental climate space has been fully captured by characterizing realized climate space, for the reasons given above.

Model averaging is a form of meta-analysis that provides some protection against the most egregious errors in model construction (e.g., inclusion of the Aleutian Islands as suitable for a giant heliophilic semi-tropical snake). However, in the absence of an appropriate characterization of fundamental climate space, there may be no objective basis for weighting competing models to be averaged by their relative merits. If unweighted averaging is conducted, the average outcome will simply reflect the distribution of models chosen by the modeler for inclusion. If all models are biased by collection locality biases or inappropriately targeting realized climate space, model averaging may not reduce the shared bias.

Our second major concern about characterizing a fundamental climate space is that MaxEnt effectively assumes that the central tendency of native range localities is an unbiased way to characterize the realized climate space boundaries. If there are more documented presence localities exhibiting low variability in daily temperature range (compared to background), for example, MaxEnt will judge low variability to be a feature indicating high suitability. This approach is untested and indirect, and in conflict with the conclusion that different factors limit distribution in different parts of the range [50]. It may be that the best way to estimate the boundaries is to find the central tendency and include all conditions within a specified threshold distance from this archetype (the MaxEnt approach, which we call “conical” because in some sense it assumes a central peak surrounded by concentrically declining suitability). Alternately, it may be that the central tendency expressed by museum locations is severely off center with regard to the fundamental climate space, with optimal climate space conditions prevailing at the edge of the occupied conditions. Asymmetry might be especially likely if a dispersal barrier prevents the species from spreading in the desired direction, or the native range is skewed by biotic factors such as competitive exclusion coming from only one side. Or the response surface may have local optima and therefore lack a central mode. Tabulating the number of presences in each condition may not reveal this deep structure, insofar as collection localities are not a random sample of suitable climate (see below). Our approach directly probes the climatic limits of the native range rather than inferring them from the central tendency of presence localities. Treating each edge of the native range as an element of interest also increases the sample size of information that can contribute to fundamental climate space characterization.

Unless constrained by regularization parameter values or user-defined constraints, MaxEnt is capable of fitting climate functions that accommodate different thresholds for different parts of the climate space. For example, if a temperature function was a square waveform with low suitability below 10 C and above 25 C, it would be plausible for the climate match limit at high latitudes to be associated with the 10 C isobar and that at low latitudes to be truncated the 25 C isobar. In the case of the climate model generated by Pyron et al., however, such dual thresholds were not present. The key climate functions showed maximal suitability at one end of the spectrum of conditions. We don't know how often this single optimum is manifest in MaxEnt climate models for wide ranging species such as the Indian Python, but its occurrence with this species undermines our confidence that MaxEnt is characterizing climate suitability appropriately at different edges of the range [50].

The radical shifts we observed in climate match with different choices of background conditions may be related to MaxEnt's reliance on collection localities (as applied in MaxEnt by Pyron et al. and many others). Several authors have highlighted the biases inherent in collection localities [30], [57]. After publication of Pyron et al., MaxEnt developer Phillips [58] suggested the pool of museum specimen collection sites where the focal species was not collected (non-collection sites, a technique known also as “target-group background” [38]) be used to characterize background with MaxEnt. Pyron et al. did not did not have the opportunity to apply this correction tool, so we cannot evaluate it on the basis of their model. However, we are skeptical that it would solve the problem of collection site bias for low-detectability species such as the Indian Python. Collection locality biases are especially severe in cryptic and wide-ranging top predators such as the Indian Python. These snakes are rarely seen; Reed and Rodda [59] reported that radiotracked pythons in Florida are seen by someone other than the radiotracker an average of once per 3.5 years. The locations where they *are* seen are generally sites with high human activity (roadsides) and good visibility (mowed grass); human activity and good transmission of light are misleading attributes to associate with the fundamental niche of a top predator; they characterize species detectability, not species presence. Thus the activity of characterizing the central climate tendency of the distinction between where pythons have and have not been collected may be grossly misleading. This may account for some of the peculiarities of our MaxEnt models, five of six of which exhibited a strong association with maritime climates (Figs. 2,5,6,8,9). Until recently, collection of museum specimens of giant constrictors was probably biased towards localities which were readily accessible, and from which it would be easier to ship giant specimens. The clustering of specimen locations near coasts, major ports, and large rivers evident from the map in Fig. 4 may account for the preponderance of MaxEnt models emphasizing maritime climates. Species distribution models that assign relative suitability by the number of specimen localities in a given climate are vulnerable to such collection site biases.

### Statistical concerns

We have four concerns regarding the typical application of MaxEnt models to invasive species climate matching: 1) the models are based on data dredging, 2) there are few restraints on overfitting, 3) the originating locations are often not statistically independent, and 4) the climate axes are not statistically independent. We believe that these four factors in aggregate contribute significantly to the model instability observed in MaxEnt models of the Indian Python (Figs. 2, 5, 6, 7, 8, 9), and may produce results with low validity.

Data dredging is the practice of drawing explicator variables blindly from a large number of possible hypotheses [60], [61]: “Running all possible models is a thoughtless approach and runs the high risk of finding effects that are, in fact, spurious if only a single model is chosen for inference” [62]. In the Pyron et al. example, the large number of climate axes, the lack of restraint on how each axis might be fitted (Pyron et al.'s fit for mean temperature of the wettest quarter and precipitation seasonality involve multiple local peaks and complex reversing functions; MaxEnt can perform similar gyrations for improvement in fit of any continuous axis), and the unlimited number of possible weightings of variables provide potentially thousands of plausible hypotheses about the causes of python distribution. “Spurious results are virtually certain with small *n,* a large number of explanatory variables, and an intense search…” [63]. Although this problem is widely recognized, MaxEnt does not solve it when used with the default settings.

A solution to the problem of data dredging is to consider only models that are chosen *a priori* on biological grounds [51], [62]. This was the approach in Rodda et al.; we identified mean monthly precipitation and temperature as good predictors of prey productivity (low-latitude rodent activity and recruitment are routinely positively correlated with seasonal rainfall) and python activity (subtropical reptiles normally limit activity to the warmer months), the requirement of suitable climates for each month of the active season (but we did not worry about inactive period extremes, as pythons can buffer themselves from those), and the durations of hibernation and aestivation as key limiting factors, and built a rule-based model structured on a plausible hypothesis from the known biology of the snake. In contrast, Pyron et al. dredged. Our more restrained approach was criticized by Pryon et al., who argued that we did not consider seasonal variability. This is incorrect. We examined the climate polygons (and we provided these for the reader to inspect: Rodda et al. Fig. 2, and in slightly modified form in Fig. 12 in the present work) for evidence of consistency among sites in the type and degree of seasonality. Although there were no sites lacking in seasonality within the accessible area (as there are, for example, in localities inhabited by the Brown Treesnake: [56]), we saw a wide range of degrees and types of seasonality. Some sites (e.g., Pakistan) showed long seasons of extremely dry climate, whereas some sites in southeast Asia and Sri Lanka exhibited no arid periods. Conversely, some sites (e.g., interior China) showed wide swings in seasonal temperature while maintaining stable precipitation levels, whereas many monsoonal sites further south (e.g., India, Java, southeast Asia) showed minimal temperature variability concurrent with radical seasonal shifts in rainfall. Thus we did consider seasonality, but did not detect a seasonality signal of sufficient consistency to merit inclusion in our model. In the absence of evidence to the contrary, we judged it prudent to include the four necessary axes, but no others, especially as additional axes were highly likely to be collinear with those already adopted (see below). Pyron et al. objected to our model because only two climate axes were included (actually, we used four, mean monthly precipitation and temperature, plus duration of hibernation and duration of aestivation, none of which Pyron et al. considered). Unlike Pyron et al. and most MaxEnt users, our axes were chosen *a priori*, to prevent data dredging. There is nothing about the structure of MaxEnt that dictates unconstrained data dredging, but default use of all available climate axes in MaxEnt constitutes data dredging.

Pyron et al. asserted that our rule based method does not consider statistical interactions among the variables. Ours did by considering not just the rectangle bounding climatically suitable localities, but the minimum convex polygon. Thus although mean monthly rainfalls of 1000 mm/month are within the range of conditions occupied by Indian Pythons in their native range, and monthly mean temperatures of 10°C are also occupied, there are no occupied sites with that combination of conditions, and any places bearing that combination would be judged unsuitable under the interaction rule of our model. One does not need to use MaxEnt to invoke ecologically relevant statistical interactions.

MaxEnt does not invoke statistical protections such as AIC against overfitting the specific data set under consideration [62], [64]. Burnham and Anderson [62] also point out that data dredging causes overfitting. Pyron et al. did not address the overfitting problem in their model. Phillips and Dudík [38] expressly state that application of MaxEnt to projection of potential invasion areas should invoke overfitting protections (regularization parameter values) stronger than those that are the MaxEnt default (used by Pyron et al.). Other lines of evidence for overfitting include the extreme model instability, and the projection of highly fragmented patches of habitat suitability within relatively climatically uniform sites such as the northwest lowland Amazon basin (e.g., Fig. 2), which contrasts with the broad areas of continuous habitat historically occupied by this top predator in Asia.

Overfitting is not only undesirable in its own right, but it complicates other issues that arise in interpretation of MaxEnt models. For example, the application of thresholds, such as minimum training presence, for geographic projections is contentious when applied to overfit models, but stable and relatively uncontroversial when applied to less complex models (e.g., AICc-MCP-90 versus AICc-MCP-86). Novel conditions are more difficult to interpret with highly parameterized models (additional areas may be denoted as novel (or unsuitable, if the user fails to make that distinction) by axes that are in actuality irrelevant), but exclusion of these areas is relatively straightforward to interpret with models of appropriate complexity. Model stability is higher, geographic matches are more stable, and the problems due to data dredging are minimized, with models of appropriate complexity.

The problem of lack of statistical independence of originating localities is a chronic problem with models like MaxEnt that identify the central tendency of an inferred climate space from the number of geographic locations in each condition [57], [65]–[67]. Pyron et al. reduced this problem by selecting only one locality point from each ∼1 km pixel, but they did not test the statistical independence that resulted, nor correct for lack of statistical independence among the pixels used [57], [58], [67]. Our model does not seek to identify a central tendency and does not weight such a choice by the number of points in each condition. Lack of independence in our localities (if it were to occur) simply results in redundant climate polygons; these do not bias our assessment of the boundaries of the aggregate climate space. Only divergent climate polygons enlarge the assessed climate space. Thus one may create species distribution models that are not dependent on statistical independence of the originating localities, but MaxEnt with default settings does not do so.

Finally, the 19 climate axes used by Pyron et al. and many other MaxEnt modelers are multi-collinear (Table S1), with the majority of axes to some degree redundant. This was particularly problematic for annual values: seven of seven precipitation axes were tightly (r>0.84) correlated with annual precipitation and nine of ten temperature axes were r>0.5 correlated with mean annual temperature (Table S1). Three pairs of correlated axes (r>0.8), annual mean temperature, minimum temperature of the coldest month, and mean temperature of the warmest quarter, all had relative variable contributions>5% to the Pyron et al. model. Multi-collinearity makes for unstable model building [62]. For example, in our recomputation of the Pyron et al. model using regional absences, two of the top three explanatory axes - constancy of temperature (Isothermality) and seasonal *in*constancy of temperature (Temperature Seasonality) – were nearly perfect inverses (r = −0.964). Inclusion of one in the model would largely negate inclusion of the other, and therefore models including both are apt to exhibit highly unstable structure with slightly varying inputs (c.f. Figs. 5,6). Pyron et al. did not address axis redundancy through axis reduction, though Pyron and Burbrink [68] did so when modeling a different species. To reduce axis redundancy one must go beyond the default settings of MaxEnt.

### Presences and absences

Pyron et al. criticized our work on the grounds that we did not use demonstrable presences, but inferred presences. We did so because demonstrable presences (museum specimen locations) are often georeferenced imprecisely (some of Pyron et al.'s localities were only recorded to the nearest degree); many collection localities for giant constrictors reference the market town in which a snake was purchased, rather than the locality in which snake was actually living), and use of demonstrable presences requires reliance on modeled climate, which can be misleading in mountainous areas [44]. As the key climate boundaries for the Indian Python are in mountainous areas (Hindu Kush, Kashmir, Himalayas, Tibetan Plateau), we worried that climate modeled at 30 arc sec (∼1 km) might reflect an average elevation that did not match the microenvironment actually occupied by Indian Pythons. These potential data inaccuracies probably had minimal influence on the aggregate climate envelope inferred, however, as demonstrated by our computation of climate space based on 84 or 98 point localities (Fig. 12). Users may judge whether uncertainty in georeferenced locality or uncertainty in climate modeling is a greater threat to model accuracy, but it appears not to have appreciably influenced the results of climate modeling of the Indian Python. Note that MaxEnt can be used with either type of locality.

One hazard of data dredging from a long list of covarying climate axes is that models with identical input localities but slightly differing background will produce substantially different climate variable importances (Fig. 10). This variability suggests that these climate variables should be treated with considerable circumspection, especially insofar as the two measures of importance given by MaxEnt were statistically uncorrelated (r^2^<0.04) in our example. The lack of correspondence is probably due to covariation among alternate axes, as suggested by the MaxEnt output warning.

One subset of the Pyron et al locality records that caught our eye was that in peninsular Thailand, south of the Isthmus of Kra and the known limit of the species. The questionable points were localities for *Python brongersmai* (Blood Python) mis-tabulated by Pyron et al. as *Python molurus* localities when extracted from the literature [41, P. Andreadis, pers. comm.]. As these points are immediately adjacent to known *Python molurus* habitat, and possess climates that are nearly identical to those occupied by *Python molurus*, one would not expect their removal to have a great impact on the inferred climate match, but they apparently shifted the weighting of axes in a consequential way (c.f. Figs. 2,5). This model sensitivity is very important in light of Pyron et al's primary conclusion that the python “…is strongly limited to the small area of suitable environmental conditions in the United States it currently inhabits…” Although the climatically suitable inland sites were relatively minor in area, the revised Overfit-Global-86 points MaxEnt model casts regulatory action in a much different light because it includes substantial areas of the U.S. (Alaska, Washington, Oregon, California, Arizona, New Mexico, Texas, Louisiana, Mississippi, Alabama, Florida, Georgia, South Carolina, and North Carolina) that are not presently inhabited by Burmese Pythons. We think MaxEnt's instability is due to the factors listed under Statistical Concerns, and it highlights the importance of careful locality selection. We also note that Pyron et al.'s assertion (see Introduction) that Indian Pythons in Florida require Everglades-like marsh habitat is not based on their MaxEnt model, information that they provided in their paper, or information published elsewhere. It is notable that pythons in Florida have already expanded beyond the boundaries of this habitat type.

Unlike MaxEnt, our method does not produce radically different climate matches depending on the exact sample points used. Thus model instability is not an inherent property of species distribution models for this species. Inclusion of the four erroneous Blood Python points would have produced no change in our climate match, as the climate in the Blood Python area was within the perimeter of the climate space outlined for the Indian Python using our method (Rodda et al. Fig. 3 shows inclusion of the Blood Python area in our match). Thus in comparison to MaxEnt modeling with default settings, other species distribution models may be relatively stable, and are not inherently vulnerable to the overfitting, sample locality biases, and background selection uncertainties apparent in the method of Pyron et al. It is not entirely clear what factors are responsible for the MaxEnt model instability, and identification of the responsible factors would be an appropriate research priority for MaxEnt users. Model instability may be due to the insensitivity of a wide ranging generalist predator such as the Indian Python to climate specifics, but if so, climate modelers need additional guidance on the conditions under which MaxEnt will perform well. In the absence of documented validity, it seems particularly premature to assert the *prima facie* validity of a specific MaxEnt model or to use one for even more speculative projections into a climate-changed future, as Pyron et al. and many other climate modelers have done.

## Supporting Information

Table S1 (0.06 MB DOC)

## References

[1] US Fish and Wildlife Service (2008). Injurious wildlife species; review of information concerning constrictor snakes from *Python*, *Boa*, and *Eunectes* genera.. Federal Register.

[2] Rodda GH, Jarnevich CS, Reed RN (2009, online 2008). What parts of the US mainland are climatically suitable for invasive alien pythons spreading from Everglades National Park?. Biol Invasions.

[3] Pyron RA, Burbrink FT, Guiher TJ (2008). Claims of potential expansion throughout the U.S. by invasive python species are contradicted by ecological niche models.. PLoS ONE.

[4] USARK (US Association of Reptile Keepers) (2009). Testimony on H.R. 2811, to amend Title 18, U.S. Code, to include constrictor snakes of the species *Python* genera as an injurious animal..

[5] Phillips SJ, Dudlik M, Schapire RE (2004). A maximum entropy approach to species distribution modeling..

[6] Phillips SJ, Anderson RP, Schapire RE (2006). Maximum entropy modeling of species geographic distributions.. Ecol Model.

[7] Guisan A, Zimmermann NE, Elith J, Graham CH, Phillips SJ (2007). What matters for predicting the occurrence of trees: techniques, data, or species' characteristics?. Ecol Monogr.

[8] Broennimann O, Treier UA, Müller-Schärer H, Thuiller W, Peterson AT (2007). Evidence of climatic niche shift during biological invasion.. Ecological Letters.

[9] Giovanelli JGR, Haddad CFB, Alexandrino J (2008). Predicting the potential distribution of the alien invasive American Bullfrog (*Lithobates catesbeianus*) in Brazil.. Biol Invasions 2007.

[10] Broennimann O, Guisan A (2008). Predicting current and future biological invasions: both native and invaded ranges matter.. Biol Lett.

[11] Rödder D, Lötters S (2009). Niche shift versus niche conservatism? Climatic characteristics of the native and invasive ranges of the Mediterranean House Gecko (*Hemidactylus turcicus*).. Global Ecol Biogeogr.

[12] Peterson AT (2006). Uses and requirements of ecological niche models and related distributional models.. Biodiversity Informatics.

[13] Phillips BL, Chipperfield JD, Kearney MR (2008). The toad ahead: challenges of modelling the range and spread of an invasive species.. Wildl Res.

[14] Elith J, Kearney M, Phillips SJ (2010). The art of modelling range-shifting species..

[15] Kearney M (2006). Habitat, environment and niche: what are we modelling?. Oikos.

[16] Kearney M, Phillips BL, Tracy CR, Christian KA, Betts G (2008). Modelling species distributions without using species distributions: the Cane Toad in Australia under current and future climates.. Ecography.

[17] Soberón J, Peterson AT (2005). Interpretation of models of fundamental ecological niches and species' distributional areas.. Biodiversity Informatics.

[18] Soberón J, Nakamura M (2009). Niches and distributional areas: concepts, methods, and assumptions.. Proc Natl Acad Sci USA.

[19] Araújo MB, Pearson RG (2005). Equilibrium of species' distributions with climate.. Ecography.

[20] Darwin C (1859). The origin of species..

[21] Soberón J (2007). Grinnellian and Eltonian niches and geographic distribution of species.. Ecol Lett.

[22] Hutchinson GE (1957). Concluding remarks.. Cold Spring Harbor Symp Quant Biol.

[23] Peterson AT, Soberón J, Sánchez-Cordero V (1999). Conservatism of ecological niches in evolutionary time.. Science.

[24] Jimenez-Valverde A, Lobo JM, Hortal J (2008). Not as good as they seem: the importance of concepts in species distribution modeling.. Divers Distrib.

[25] Jeschke JM, Strayer DL (2008). Usefulness of bioclimatic models for studying climate change and invasive species.. Ann N Y Acad Sci.

[26] Colautti RI, Ricciardi A, Grigorovich IA, MacIsaac HJ (2004). Is invasion success explained by the enemy release hypothesis?. Ecol Lett.

[27] Duncan RP, Cassey P, Blackburn TM (2009). Do climate envelope models transfer? A manipulative test using dung beetle introductions.. Proc Roy Soc Lond B.

[28] Randin CF, Dirnböck T, Dullinger S, Zimmermann NE, Zappa M (2006). Are niche-based species distribution models transferable in space?. J Biogeogr.

[29] Peterson AT, Papes M, Eaton M (2007). Transferability and model evaluation in ecological niche modeling: a comparison of GARP and Maxent.. Ecography.

[30] Phillips SJ (2008). Transferability, sample selection bias and background data in presence-only modelling: a response to Peterson et al. (2007).. Ecography.

[31] Beaumont LJ, Gallagher RV, Thuiller W, Downey PO, Leishman MR (2009). Different climatic envelopes among invasive populations may lead to understimations of current and future biological invasions.. Divers Dist.

[32] Peterson AT, Vieglais DA (2001). Predicting species invasions using ecological niche modeling: new approaches from bioinformatics attack a pressing problem.. Bioscience.

[33] Fitzpatrick MC, Weltzin JF, Sanders NJ, Dunn RR (2007). The biogeography of prediction error: why does the introduced range of the fire ant over-predict its native range?. Global Ecol Biogeogr.

[34] Loo SE, Mac Nally RC, Lake PS (2007). Forecasting New Zealand mudsnail invasion range: model comparisons using native and invaded ranges.. Ecol Appl.

[35] Lobo JM, Jimenez-Valverde A, Real R (2008). AUC: a misleading measure of the performance of predictive distribution models.. Glob Ecol Biogeog.

[36] Beaumont LJ, Hughes L, Poulsen M (2005). Predicting species distributions: use of climatic parameters in BIOCLIM and its impact on predictions of species' current and future distributions.. Ecol Model.

[37] Heikkinen RK, Luoto M, Araújo MB, Virkkala R, Thuiller W (2006). Methods and uncertainties in bioclimatic envelope modelling under climate change.. Progress in Physical Geography.

[38] Phillips SJ, Dudík M (2008). Modeling of species distributions with Maxent: new extensions and comprehensive evaluation.. Ecography.

[39] Warren DL, Seifert SN (2010). Environmental niche modeling in Maxent: the importance of model complexity and the performance of model selection criteria..

[40] VanDerWal J, Shoo LP, Graham C, Williams SE (2009). Selecting pseudo-absence data for presence-only distribution modeling: how far should you stray from what you know?. Ecol Model.

[41] Nabhitabhata J, Chan-ard T (2005). Status of mammals, reptiles and amphibians in Thailand..

[42] Lobo JM (2008). More complex distribution models or more representative data?. Biodiversity Informatics.

[43] Lobo JM, Jimenez-Valverde A, Hortal J (2010). The uncertain nature of absences and their importance in species distribution modelling.. Ecography.

[44] Hijmans RJ, Cameron SE, Parra JL, Jones PG, Jarvis A (2005). Very high resolution interpolated climate surfaces for global land areas.. International Journal of Climatology.

[45] Deyang L (1986). *Python molurus bivittatus* occurred in Qingchuan County of Sichuan Province [in Chinese].. Acta Herpetologica Sinica.

[46] Khan MS (2002). A guide to the snakes of Pakistan..

[47] Khan MS (2006). Amphibians and reptiles of Pakistan..

[48] Mertens R (1969). Die Amphibien und Reptilien West-Pakistans.. Stuttgarter Beiträge zur Naturkunde aus dem Staatlichen Museum für Naturkunde in Stuttgart.

[49] Minton SA (1966). A contribution to the herpetology of West Pakistan.. Bull Amer Mus Natur Hist.

[50] Brown JH, Stevens GC, Kaufman DM (1996). The geographic range: size, shape, boundaries, and internal structure.. Annu Rev Ecol Syst.

[51] Araújo MB, Guisan A (2006). Five (or so) challenges for species distribution modelling.. J Biogeogr.

[52] Rödder D, Lötters S (2010). Potential distribution of the alien invasive Brown Tree Snake, *Boiga irregularis* (Reptilia: Colubridae).. Pac Sci.

[53] Rödder D, Solé M, Böhme W (2008). Predicting the potential distribution of two alien invasive housegeckos (Gekkonidae: *Hemidactylus frenatus*, *Hemidactylus mabouia*).. North-West J Zool.

[54] Martin WK (1996). The current and potential distribution of the Common Myna *Acridotheres tristis* in Australia.. Emu.

[55] Zambrano L, Martínez-Meyer E, Menezes N, Peterson AT (2006). Invasive potential of common carp (*Cyprinus carpio*) and Nile tilapia (*Oreochromis niloticus*) in American freshwater systems.. Can J Fish Aquat Sci.

[56] Rodda GH, Reed RN, Jarnevich CS, Witmer GW, Pitt WC, Fagerstone KA (2007). Climate matching as a tool for predicting potential North American spread of Brown Treesnakes.. Managing Vertebrate Invasive Species: proceedings of an international symposium.

[57] Veloz SD (2009). Spatially autocorrelated sampling falsely inflates measures of accuracy for presence-only nich models.. J Biogeogr.

[58] Phillips SJ, Dudìk M, Elith J, Graham CH, Lehmann A (2009). Sample selection bias and presence-only distribution models: implications for background and pseudo-absence data.. Ecol Appl.

[59] Reed RN, Rodda GH (2009). Giant constrictors: biological and management profiles and an establishment risk assessment for nine large species of pythons, anacondas, and the Boa Constrictor.. US Geological Survey Open File Report.

[60] Freedman DA (1983). A note on screening regression equations.. American Statistician.

[61] Rexstad EA, Miller DD, Flather CH, Anderson EM, Hupp JW (1988). Questionable multivariate statistical inference in wildlife habitat and community studies.. J Wildl Manage.

[62] Burnham KP, Anderson DR (2002). Model selection and multimodel inference; a practical information-theoretic approach, 2nd edn..

[63] Anderson DR, Burnham KP, Gould WR, Cherry S (2001). Concerns about finding effects that are actually spurious.. Wildl Soc Bull.

[64] Wisz MS, Guisan A (2009). Do pseudo-absence selection strategies influence species distribution models and their predictions?.

[65] Guisan A, Zimmermann NE (2000). Predictive habitat distribution models in ecology.. Ecol Model.

[66] Segurado P, Araújo MB, Kunin WE (2006). Consequences of spatial autocorrelation for niche-based models.. J Appl Ecol.

[67] Dormann CF, McPherson JM, Araújo MB, Bivand R, Bolliger J (2007). Methods to account for spatial autocorrelation in the analysis of species distributional data: a review.. Ecography.

[68] Pyron RA, Burbrink FT (2009). Lineage diversification in a widespread species: roles for niche divergence and conservatism in the common kingsnake, *Lampropeltis getula*.. Mol Ecol.

[69] Thorton PE, Running SW, White MA (1997). Generating surfaces of daily meteorology variables over large regions of complex terrain.. J Hydrol.

