# Supplementary material for: Challenges in Identifying Sites Climatically Matched to the Native Ranges of Animal Invaders
Source: PLoS One. 2011 Feb 9;6(2):e14670. doi: 10.1371/journal.pone.0014670 (PMC3036589; doi:10.1371/journal.pone.0014670)
Supplement: Table S1 — (0.06 MB DOC) [file pone.0014670.s001.doc]

**Table S1.** Correlations among climate axes used in MaxEnt, as illustrated by 5000 localities from within the geographic area used by Pyron et al. for background points, excluding 4 peninsular Thailand sites erroneously included due to presence of Blood Pythons. Values in excess of 0.8 presented in bold.

|  | BIO1 | BIO2 | BIO3 | BIO4 | BIO5 | BIO6 | BIO7 | BIO8 | BIO9 | BIO10 | BIO11 | BIO12 | BIO13 | BIO14 | BIO15 | BIO16 | BIO17 | BIO18 | BIO19 |
| --- | --- | --- | --- | --- | --- | --- | --- | --- | --- | --- | --- | --- | --- | --- | --- | --- | --- | --- | --- |
| BIO1 | 1 |  |  |  |  |  |  |  |  |  |  |  |  |  |  |  |  |  |  |
| BIO2 | 0.511 | 1 |  |  |  |  |  |  |  |  |  |  |  |  |  |  |  |  |  |
| BIO3 | **0.834** | 0.443 | 1 |  |  |  |  |  |  |  |  |  |  |  |  |  |  |  |  |
| BIO4 | **-0.805** | -0.121 | **-0.811** | 1 |  |  |  |  |  |  |  |  |  |  |  |  |  |  |  |
| BIO5 | **0.874** | 0.743 | 0.642 | -0.428 | 1 |  |  |  |  |  |  |  |  |  |  |  |  |  |  |
| BIO6 | **0.96** | 0.312 | **0.854** | **-0.929** | 0.714 | 1 |  |  |  |  |  |  |  |  |  |  |  |  |  |
| BIO7 | -0.681 | 0.118 | -0.706 | **0.968** | -0.246 | **-0.855** | 1 |  |  |  |  |  |  |  |  |  |  |  |  |
| BIO8 | **0.805** | 0.553 | 0.65 | -0.447 | **0.844** | **0.678** | -0.312 | 1 |  |  |  |  |  |  |  |  |  |  |  |
| BIO9 | **0.938** | 0.418 | 0.794 | **-0.844** | 0.762 | **0.947** | -0.745 | 0.597 | 1 |  |  |  |  |  |  |  |  |  |  |
| BIO10 | **0.923** | 0.654 | 0.682 | -0.516 | **0.987** | 0.788 | -0.359 | **0.867** | **0.812** | 1 |  |  |  |  |  |  |  |  |  |
| BIO11 | **0.976** | 0.392 | **0.867** | **-0.913** | 0.755 | **0.995** | **-0.817** | 0.712 | **0.95** | **0.821** | 1 |  |  |  |  |  |  |  |  |
| BIO12 | 0.381 | -0.165 | 0.577 | -0.513 | 0.149 | 0.472 | -0.543 | 0.263 | 0.372 | 0.219 | 0.446 | 1 |  |  |  |  |  |  |  |
| BIO13 | 0.473 | -0.028 | 0.613 | -0.543 | 0.269 | 0.529 | -0.533 | 0.393 | 0.424 | 0.332 | 0.52 | **0.902** | 1 |  |  |  |  |  |  |
| BIO14 | 0.053 | -0.309 | 0.225 | -0.214 | -0.113 | 0.152 | -0.294 | -0.056 | 0.101 | -0.06 | 0.113 | 0.718 | 0.414 | 1 |  |  |  |  |  |
| BIO15 | 0.385 | 0.553 | 0.329 | -0.157 | 0.471 | 0.266 | -0.019 | 0.475 | 0.269 | 0.45 | 0.321 | -0.152 | 0.155 | -0.486 | 1 |  |  |  |  |
| BIO16 | 0.458 | -0.05 | 0.608 | -0.541 | 0.248 | 0.52 | -0.536 | 0.37 | 0.416 | 0.311 | 0.509 | **0.926** | **0.993** | 0.448 | 0.109 | 1 |  |  |  |
| BIO17 | 0.081 | -0.303 | 0.26 | -0.243 | -0.093 | 0.183 | -0.321 | -0.033 | 0.129 | -0.038 | 0.143 | 0.755 | 0.453 | **0.993** | -0.482 | 0.488 | 1 |  |  |
| BIO18 | 0.222 | -0.131 | 0.371 | -0.296 | 0.056 | 0.264 | -0.324 | 0.265 | 0.145 | 0.118 | 0.253 | **0.8** | 0.762 | 0.564 | -0.082 | 0.776 | 0.587 | 1 |  |
| BIO19 | 0.254 | -0.185 | 0.441 | -0.382 | 0.076 | 0.352 | -0.431 | 0.078 | 0.311 | 0.126 | 0.315 | 0.76 | 0.578 | 0.69 | -0.267 | 0.607 | 0.72 | 0.389 | 1 |
